# Supplementary material for: OcuPair, a Novel Photo‐crosslinkable PAMAM Dendrimer‐Hyaluronic Acid Hydrogel Bandage/Bioadhesive for Corneal Injuries and Temporary Corneal Repair
Source: Adv Sci (Weinh). 2025 Mar 27;12(23):2417731. doi: 10.1002/advs.202417731 (PMC12199432; doi:10.1002/advs.202417731)
Supplement: Supplementary file 1 — Supporting Information [file ADVS-12-2417731-s004.pdf]

ADVANCED SCIENCE
Open Access

Supporting Information

for *Adv. Sci.*, DOI 10.1002/advs.202417731

OcuPair, a Novel Photo-crosslinkable PAMAM Dendrimer-Hyaluronic Acid Hydrogel Bandage/Bioadhesive for Corneal Injuries and Temporary Corneal Repair

Siva P. Kambhampati, Rishi Sharma, Hui Lin, Santiago Appiani, Jeffrey L. Cleland, Samuel C. Yiu and Rangaramanujam M. Kannan**

Supporting Information

OcuPair, a novel photo-crosslinkable PAMAM dendrimer-hyaluronic acid hydrogel bandage/bio-adhesive for corneal injuries and temporary corneal repair

Siva P. Kambhampati[1,2, §], Rishi Sharma[1,2, §], Hui Lin[1, §], Santiago Appiani[2], Jeffrey L. Cleland[2], Samuel C Yiu[1*], Rangaramanujam M. Kannan[1,2*]

[1]Center for Nanomedicine, Wilmer Eye Institute, Department of Ophthalmology, Johns Hopkins University School of Medicine, Baltimore, MD, USA

[2] Ashvattha Therapeutics, Inc., Baltimore, MD, USA.

[§] Co-first authors with equal contribution to this work.

*Corresponding authors:

Rangaramanujam M. Kannan, Professor of Ophthalmology, Center for Nanomedicine at the Wilmer Eye Institute, 400 North Broadway, Baltimore, Maryland 21231, USA. e-mail: krangar1@jhmi.edu

Samuel C Yiu, Associate Professor of Ophthalmology, Wilmer Eye Institute, 400 North Broadway, Baltimore, Maryland 21231, USA. e-mail: syiu2@jhmi.edu

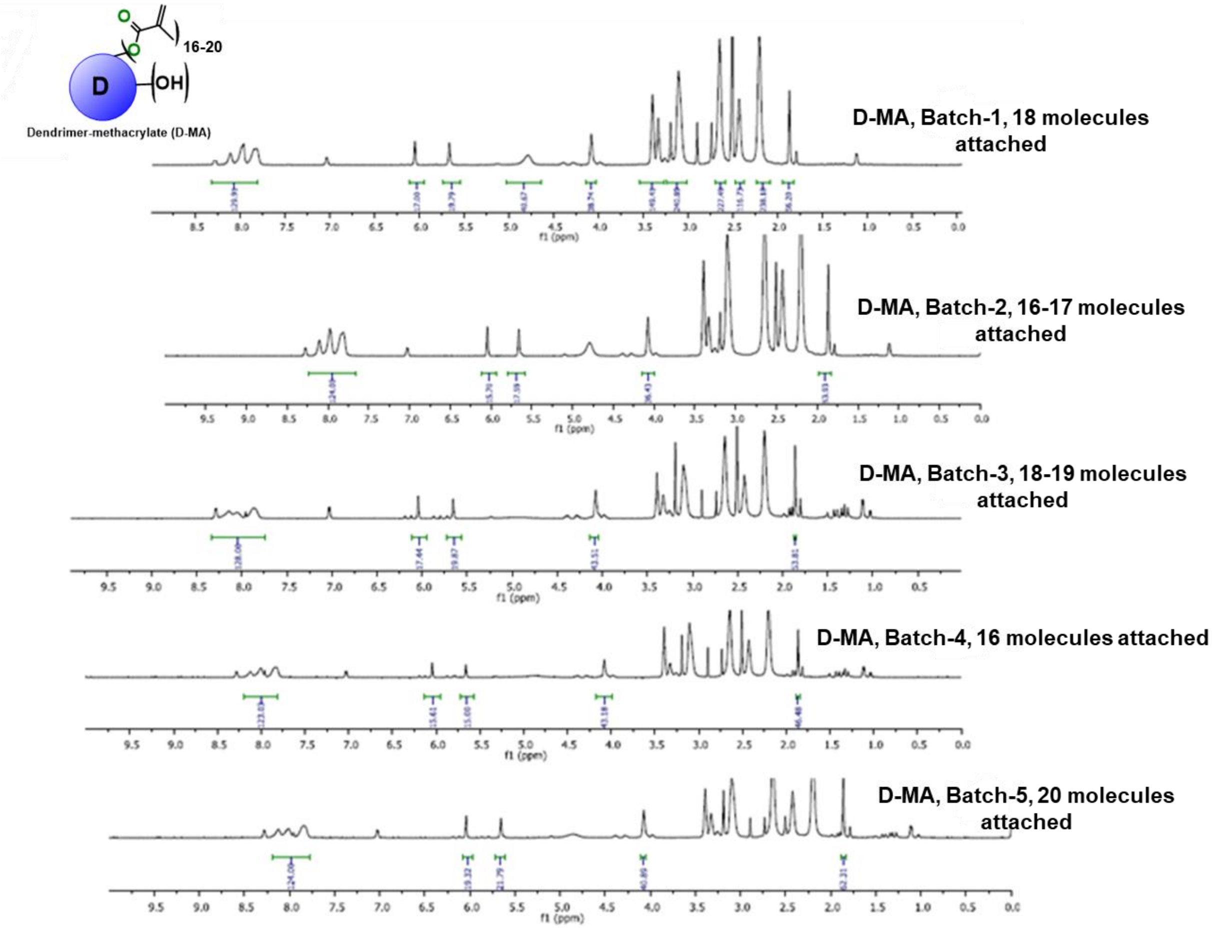

Figure S1: ^{1}H NMR spectra of different batches of D-MA synthesized using the optimized protocol reported demonstrating consistent methacrylate loading.

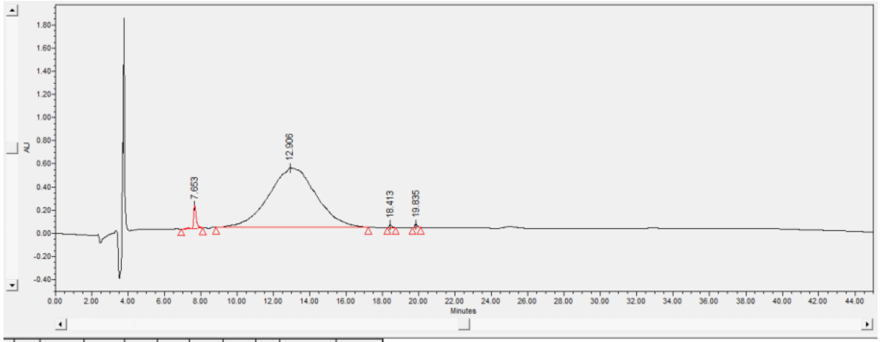

	Name	Retention Time (min)	Area (μV*sec)	% Area	Height (μV)	Int Type	Amount	Units	Peak Type	Peak Codes
1		7.653	2020779	2.13	195824	bb			Unknown	
2		12.906	92438673	97.27	514835	bb			Unknown	
3		18.413	256525	0.27	23950	bb			Unknown	
4		19.835	317881	0.33	29672	bb			Unknown	

Figure S2: HPLC chromatogram of synthesized D-MA conjugate demonstrating a purity >95% eluting at 12.9 min.

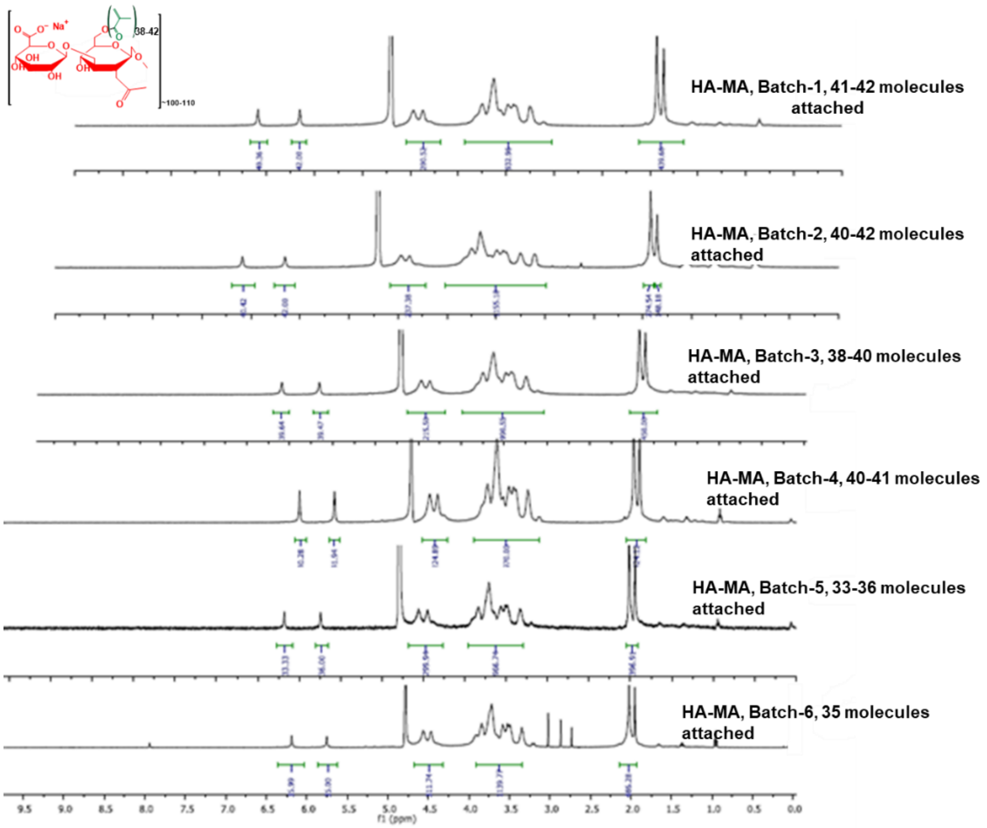

Figure S3: ^{1}H NMR spectra of different batches of HA-MA synthesized using the optimized protocol reported demonstrating consistent methacrylate loading.

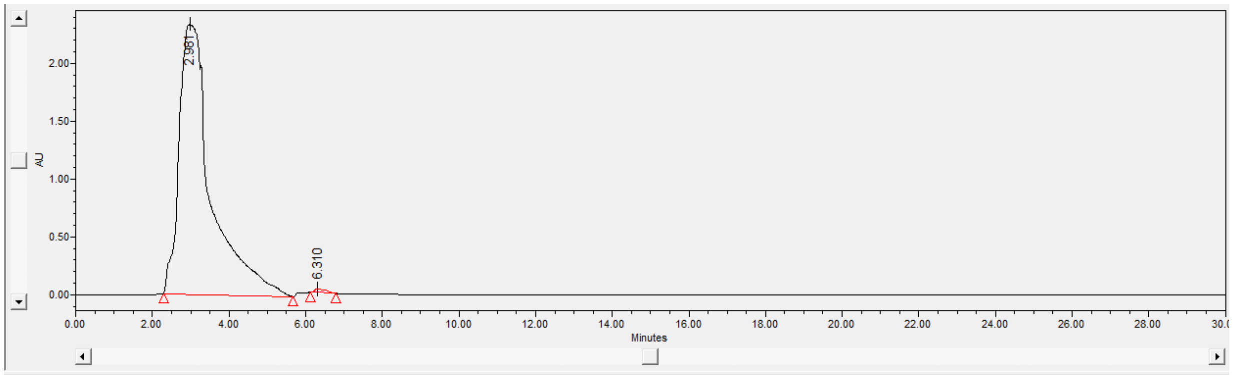

	Name	Retention Time (min)	Area (µV*sec)	% Area	Height (µV)	Int Type	Amount	Units	Peak Type	Peak Codes
1		2.981	134669709	99.52	2330408	bb			Unknown	
2		6.310	647116	0.48	33283	bb			Unknown	I08

Figure S4: HPLC chromatogram of synthesized HA-MA conjugate demonstrating purity >97% eluting at 2.98 min.

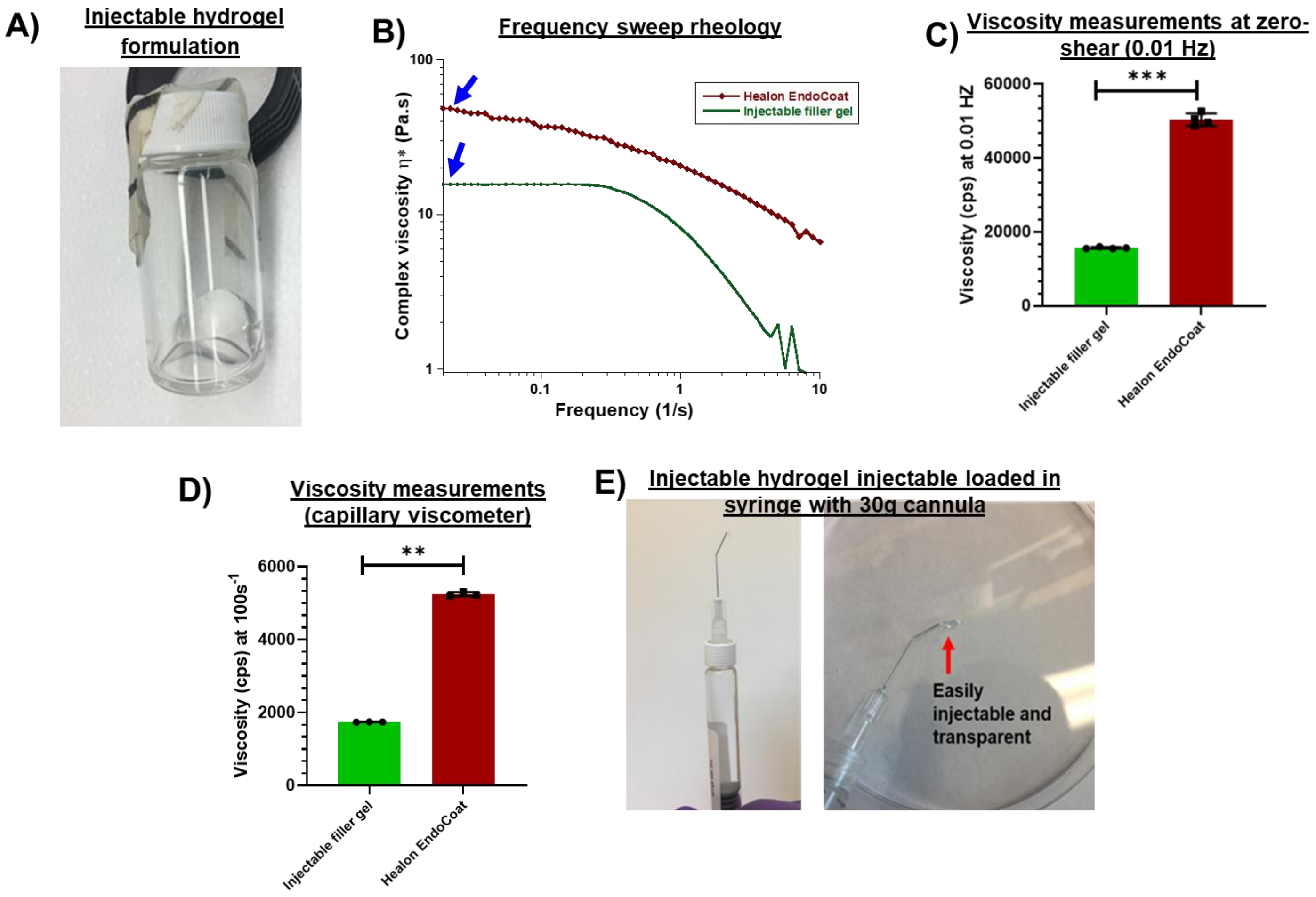

Figure S5: Viscosity measurements of OcuPair injectable hydrogel formulation. A) Image of injectable hydrogel after sterilization demonstrating that the solution is transparent and viscous. **B)** Dynamic frequency sweep rheology of injectable hydrogel and using parallel plate rheometer demonstrating shear thinning properties at low viscosity compare the Healon Endocoat. **C)** Complex viscosity measurements at zero shear frequency of 0.01 Hz for injectable hydrogel and compared with Healon Endocoat. **D)** Viscosity of Injectable hydrogel formulation at constant frequency of 100 s^{-1} using capillary viscometer. **E)** Image of injectable hydrogel loaded into a glass syringe fitted with 30G anterior chamber cannula demonstrating that the injectable hydrogel can be easily extruded through narrow gauge needle.

Table S1: Summary of findings in the process of optimizing the OcuPair adhesive hydrogel formulation.

**D-MA: HA-MA ratio in the formulation**	**Viscosity (cps)**	**Gelation time (seconds)**	**Gel characteristics and appearance**	**Residence time after applying on the cornea**	**Burst pressure in rabbit eyes (mmHg) for linear incision**	**Image**
100% D-MA (100:0)	~96.8 ± 6.6 (n=7)	~10-20 s	Forms thin and brittle gels and the gel breaks when twisted using forceps	The solution flows away or flows into the corneal incision immediately applying	NA	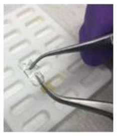
100% HA-MA (0:100)	~7306.5 ± 336.5 (n=12)	>200 s	Forms patchy weak gels	The solution stays on the corneal incision for more than 20 s after application	NA	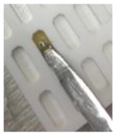
70:30	~4142.3 ± 185 (n=6)	~30-45 s	Forms brittle gels and gels breaks when handled	The solution stays on corneal surface for ~10-15s	~42.8 ± 7.2 mmHg The hydrogel layer peels from corneal surface quickly at burst pressure	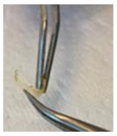
50:50	~5068 ± 87 (n=6)	~30-45 s	Forms flexible gels but some gels breaks when handled	The solution stays on corneal surface for ~15-20 s after application	~58.4 ± 6.8 mmHg The hydrogel layer peels from corneal surface at burst pressure	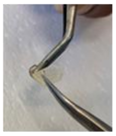
30:70	~5205.8 ± 190 (n=6)	~60-80 s	Forms flexible and transparent and sticky gels and can be easily handled using instruments	The solution stays on corneal surface for ~15-20 s upon application	~78.4 ± 2.6 mmHg. The hydrogel bandage peels from the corneal surface at burst pressure but withstands ~60mmHg for 3-5 mins	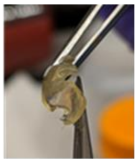

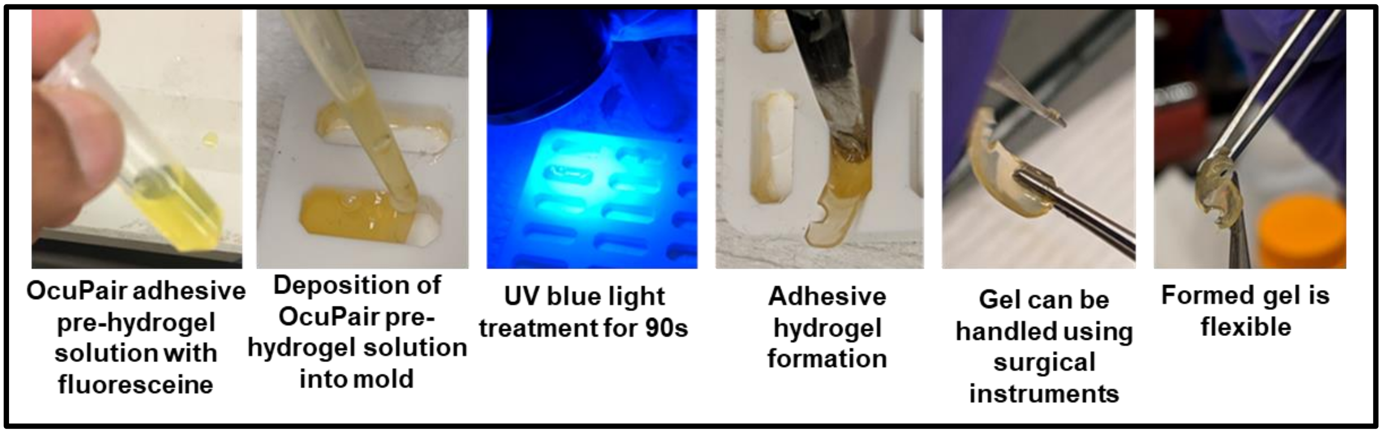

Figure S6: Photographs depicting the stepwise procedure for preparation of the adhesive hydrogel. The adhesive hydrogel precursor solution (with 0.02% fluorescein) filled in the TEM molds and photo-crosslinked using cobalt blue UV light for 90 seconds. The flexibility of the adhesive hydrogel was demonstrated by manipulating it with the surgical forceps. The adhesive hydrogel is transparent which is evident from the glass slide experiment

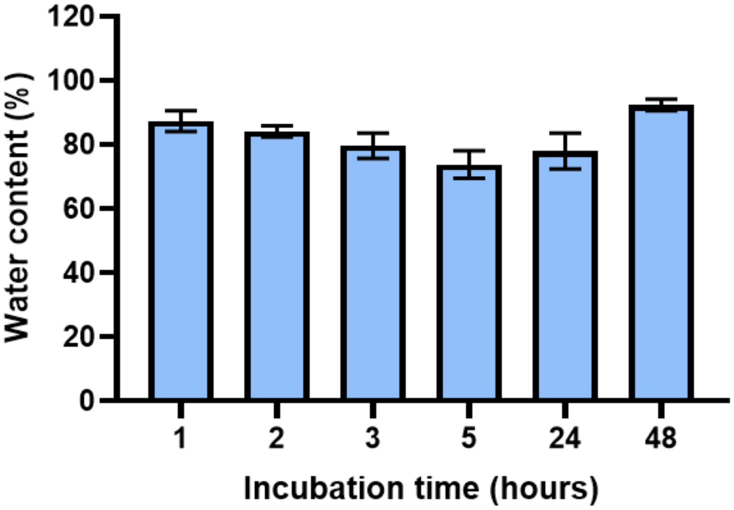

Figure S7: Evaluation of degree of hydration or water content in OcuPair adhesive hydrogel (30: 70, D-MA: HA-MA) in simulated tear fluid.

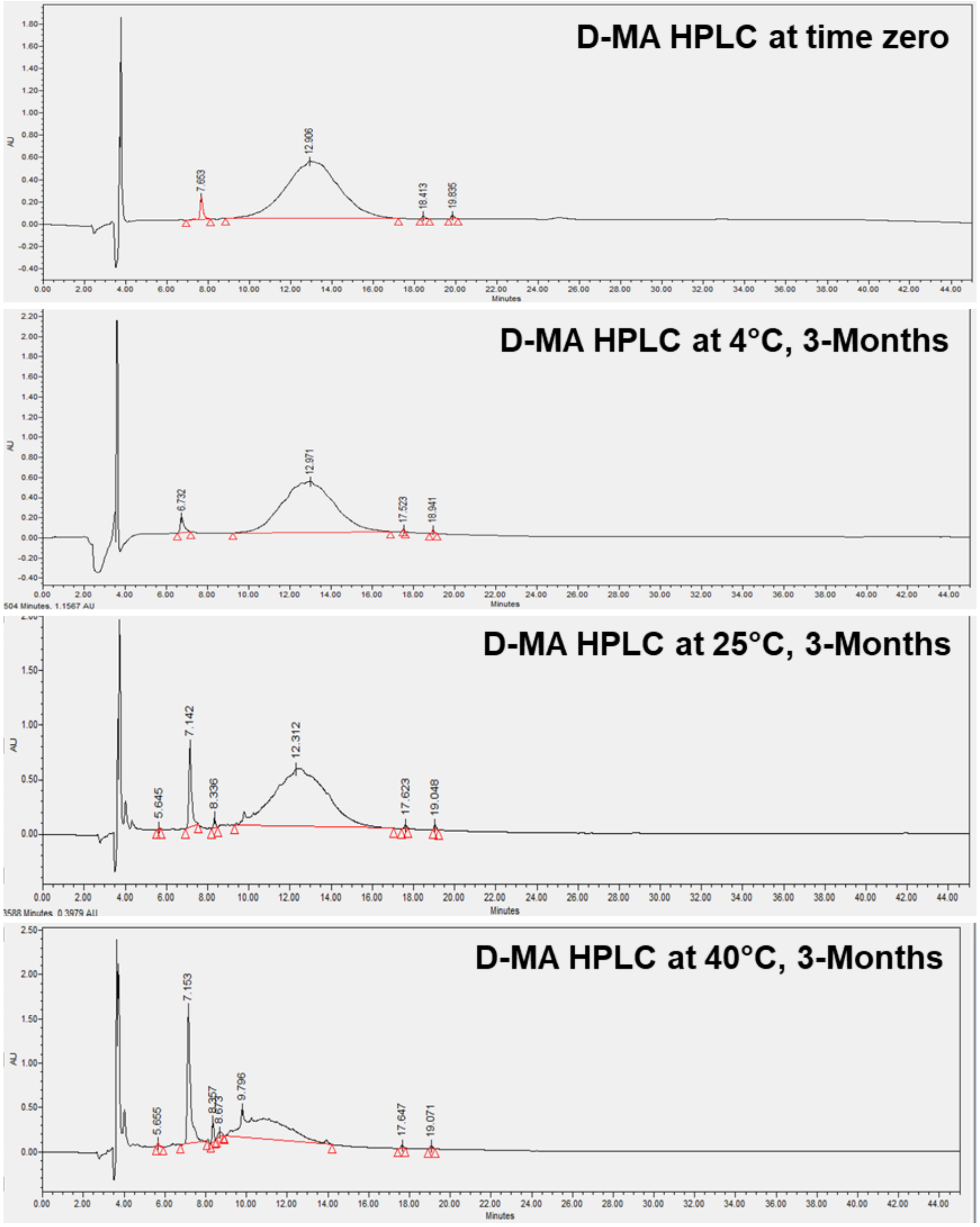

Figure S8: Stability evaluation of D-MA solution using HPLC. HPLC chromatograms of D-MA (300 mg/mL) solution in phosphate buffer (pH 6) stored at 4ºC, 25ºC, and 40ºC at 3-month time point compared with time zero.

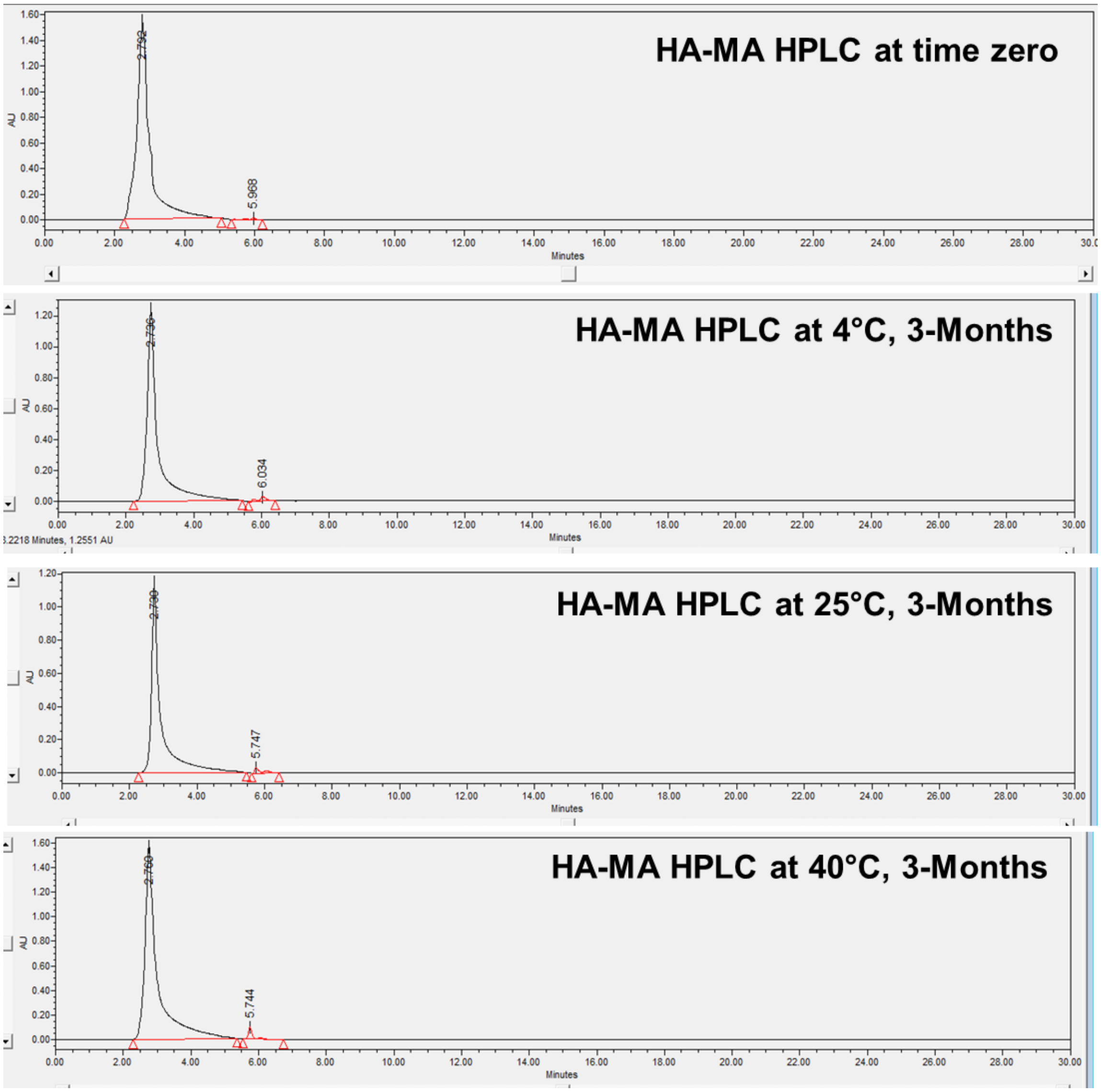

Figure S9: Stability evaluation of HA-MA solution using HPLC. HPLC chromatograms of HA-MA (190 mg/mL) solution in phosphate buffer (pH 6) stored at 4ºC, 25ºC, and 40ºC at 3-month time point compared with time zero.

Summary of stability evaluation of Dendrimer-Methacrylate (D-MA) solution (300mg/mL)									
Time points/Temperature	4°C			25°C			40°C		
Parameters	HPLC purity (%)	pH	Osmolality (moSm/kg)	HPLC purity (%)	pH	Osmolality (moSm/kg)	HPLC purity (%)	pH	Osmolality (moSm/kg)
Time Zero	97.3	7.59	201						
1 week	97.5	7.37	208	96.8	7.42	237	94.8	7.40	300
1 month	97.6	7.39	200	95.6	7.48	236	78.7	7.31	382
2 months	97.6	7.37	215	92.2	7.35	255	74.3	7.63	396
3 months	97.3	7.31	205	92.3	7.47	249	65.4	7.23	356

Summary of stability evaluation of Hyaluronic acid-Methacrylate (HA-MA) solution (190mg/mL)									
Time points/Temperature	4°C			25°C			40°C		
Parameters	HPLC purity (%)	pH	Osmolality (moSm/kg)	HPLC purity (%)	pH	Osmolality (moSm/kg)	HPLC purity (%)	pH	Osmolality (moSm/kg)
Time Zero	99.5	7.61							
1 week	99.3	7.42		99.6	7.34		99.0	7.59	
1 month	99.2	7.37		98.6	7.61		98.5	7.43	
2 months	98.4	7.55		98.9	7.54		98.2	7.48	
3 months	98.3	7.39		98.0	7.29		98.0	7.67	

Table S2: Summary of stability evaluation if D-MA and HA-MA up to 3-month timepoint at different storage temperatures.

Table S3: Summary of *ex vivo* eye burst pressure measurements in rabbit and porcine eyeballs.

**Summary of ex vivo eye burst pressure measurements**		
**Incision Type**	**Porcine Eyes (mature)**	**Rabbit eyes (mature)**
**Linear full thickness (5-6mm)**	101.5 ± 6.5 mmHg (n=18)	78.4 ± 2.6 mmHg (n=16)
**Stellate (three pronged) (3.5 mm each prong)**	92.6 ± 5.8 mmHg (n=18)	74.7± 5.4 mmHg (n=16)
**Circular incision with tissue present (4.5mm)**	84.2 ± 9.3 mmHg (n=18)	58.4 ± 3.0 mmHg (n=12)
**Corneal perforations (3mm dia)**	115 ± 5.3 mmHg (n=16)	86.2 ± 7.2 mmHg (n=13)
**Corneal-scleral incisions 6mm horizontal and 4mm vertical**	>120 mmHg (n=18)	>100 mmHg (n=15)

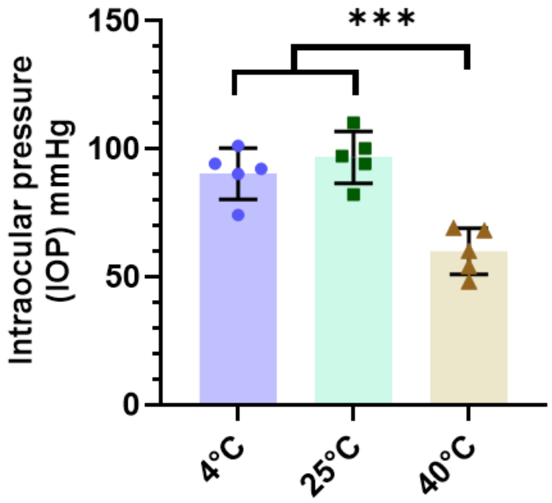

Figure S10: Evaluation of effect of D-MA degradation during stability studies on burst pressure measurements in ex vivo porcine eyes. The adhesive hydrogel formulation was prepared by using the D-MA solution (300mg/mL) stored for 3 months at 4°C, 25°C and 40°C.

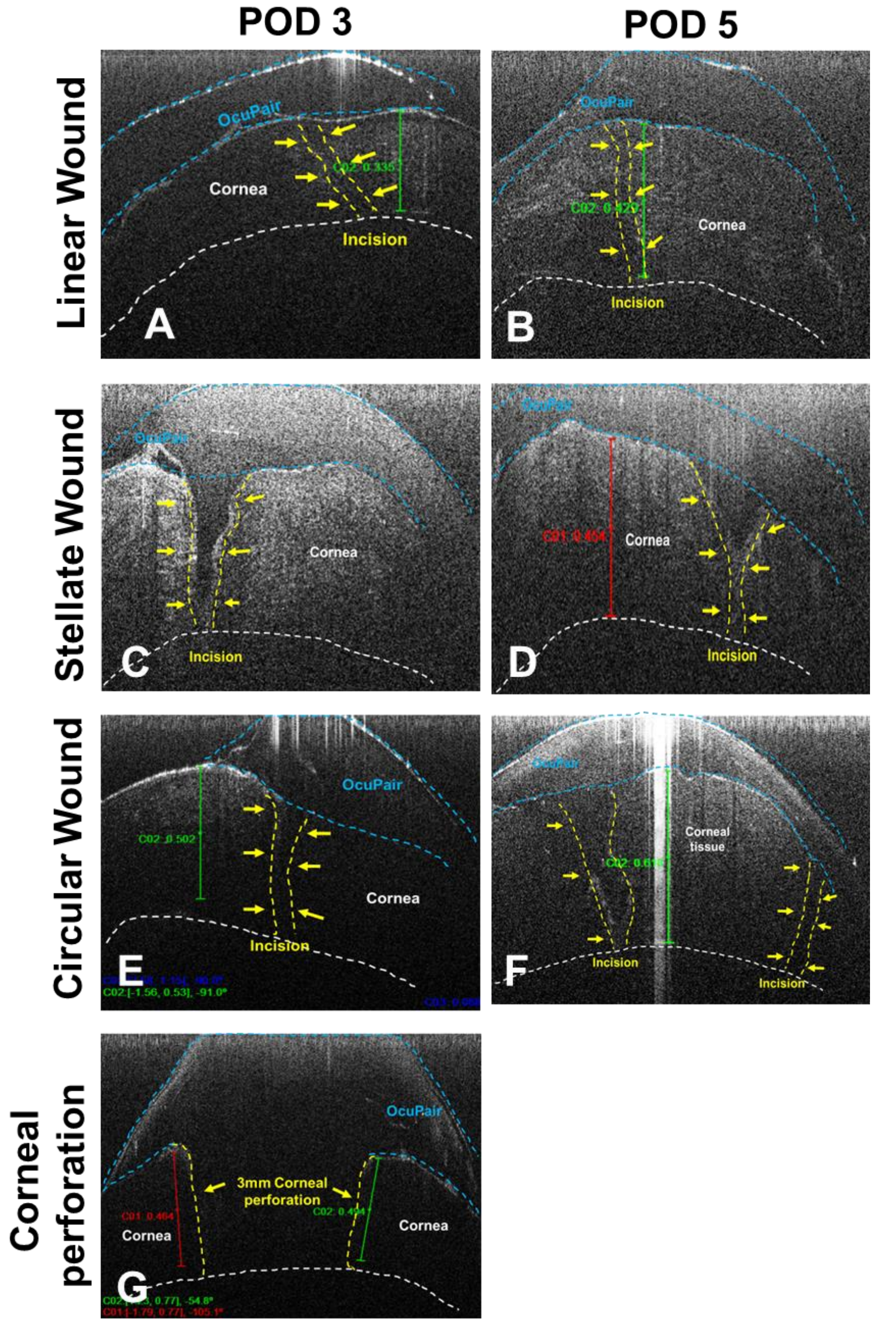

Figure S11: Anterior segment optical coherence tomography (AS-OCT) imaging of the rabbit corneas with different full thickness wounds sealed using OcuPair adhesive hydrogel at POD3 and POD5. The cornea is delineated using white broken lines, the incision edges are delineated in yellow and arrows, and the OcuPair adhesive hydrogel bandage is indicated in blue.

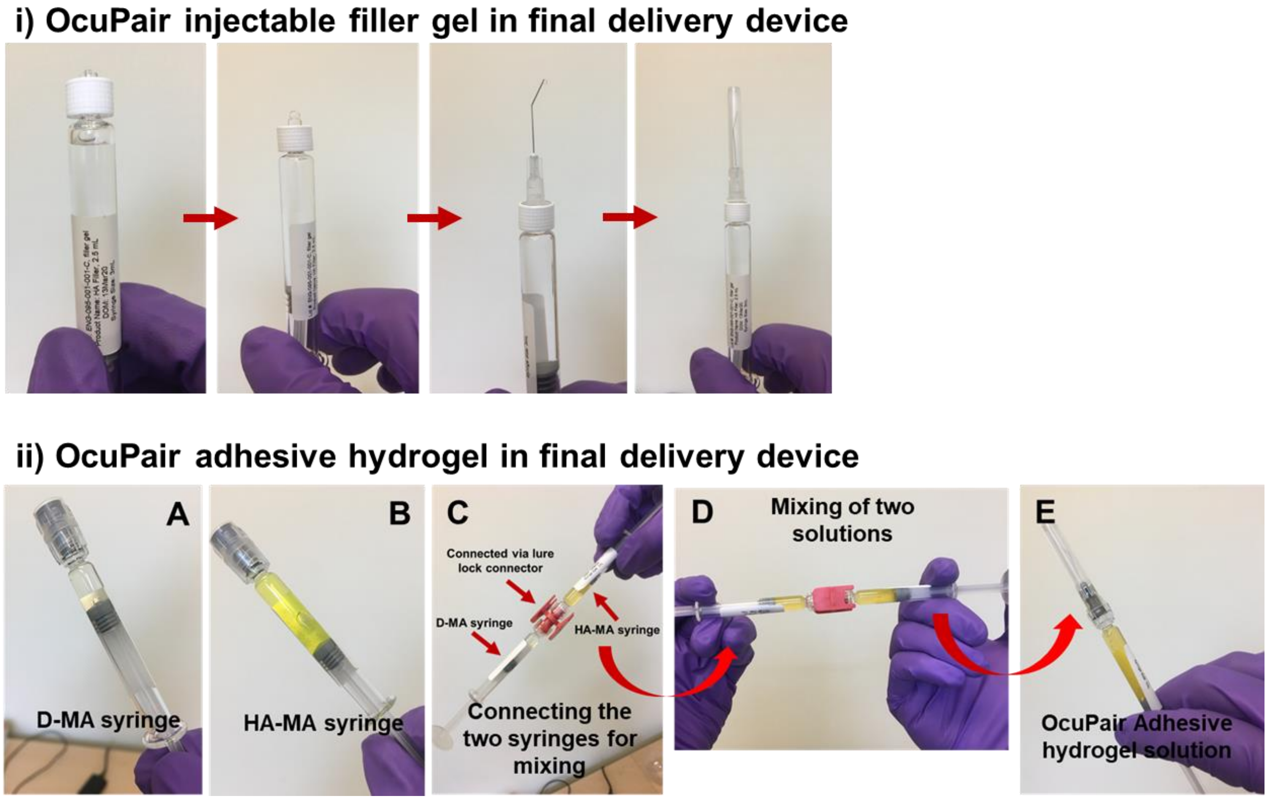

Figure S12: OcuPair injectable hydrogel and adhesive hydrogel pre-formulation loaded into final delivery devices as part of OcuPair kit. (i) OcuPair injectable hydrogel (sterilized) loaded into 3mL glass syringes with lure-lok connector under sterile conditions. A 30G anterior chamber cannula cane connected to the syringe and the injectable hydrogel can be easily injected into the intraocular cavity. **(ii)** The individual components of the adhesive hydrogel D-MA (300 mg/mL) and HA-MA (190mg/mL) with 0.05% fluoresceine (hence the yellow color) were loaded in to separate 1 mL glass syringes with luer-lok caps aseptically. The two solutions are mixed by connecting the two syringes using a lure-lok connector and all the contents are pushed into one syringe and fitted with a 27G cannula for applying over the corneal wound. All the components are provided as part of OcuPair kit.

Tabel S4: Summary for GLP biosafety evaluation of OcuPair (at Charles Rivers, CRO) in accordance with "*Biological evaluation of medical devices (ISO 10993)*"

Study #	Category	Test type/name	Results and findings
3101-006-01	ISO 10993-6	Local effects after *in vivo* implantation of OcuPair adhesive hydrogel (subconjunctival) in ocular tissue (30-days study) (i) Subconjunctival injection of OcuPair adhesive hydrogel solution followed by crosslinking with UV light (ii) 4 min exposure of UV light (365nm, 3W)	Subconjunctival adhesive hydrogel persisted until day 30 and was well tolerated. Minimal to mild conjunctivitis between day 3-7 but resolved by day 10. No signs of local toxicity on day 30.<br><br>4min UV light treatment did not cause cataract, lens opacification and changes in retinal function (ERG) compared to healthy eyes.
3101-006-02	ISO 10993-6	30-day ocular toxicity (*In vivo*) study of OcuPair.<br>**(i)** Injectable hydrogel anterior, **(ii)** injectable hydrogel posterior, **(iii)** OcuPair pre-hydrogel solution anterior, **(iv)** OcuPair pre-hydrogel solution epiconjunctival, **(v)** Irgacure/Ominirad 2959 epiconjunctival and subconjunctival.	Injectable hydrogel and the components of adhesive pre-hydrogel were well tolerated with no signs of corneal and intraocular inflammation, infections/uveitis and toxicity on day 30.<br>Two animals had mild anterior lens capsule opacification on day 7 but completely resolved by day 29.
3101-005	ISO 10993-11<br>ISO 10993-12	Acute systemic toxicity of OcuPair in a mouse model	Intraperitoneal and intravenous injections of OcuPair adhesive hydrogel precursor solution did not demonstrate any signs of systemic toxicity based on body weight changes and clinical observations.
3101-004	ISO 10993-10	Intracutaneous reactivity of OcuPair in rabbits	OcuPair adhesive hydrogel extracts did not demonstrate or cause significant reactions or long-term (30 days) intradermal edema or irritation in healthy rabbits.
3101-003-01	ISO 10993-10	Skin sensitization study in Guinea pigs	The topical OcuPair adhesive hydrogel application did not demonstrate any signs of allergic contact dermatitis even after re-challenging (applied twice).
3101-003-02	ISO 10993-10	Skin irritation study in Guinea pigs	Topical OcuPair adhesive hydrogel did not demonstrate any signs of skin irritation (reddening) in healthy Guinea pigs.

9602809	ISO 10993-5	Neutral Red Uptake (NRU) Cytotoxicity Test with BALB/c 3T3 cells for OcuPair adhesive hydrogel	OcuPair adhesive pre-hydrogel solution did not demonstrate significant cytotoxicity with cell viability (~70%) and 200mg/mL and with IC_{50} >200mg/mL and <400mg/mL.
9602868	ISO 10993-3	Bacterial Reverse Mutation Test in Salmonella typhimurium and Escherichia coli for OcuPair adhesive hydrogel	OcuPair adhesive hydrogel in polar and non-polar vehicle did not demonstrate any evidence of genotoxicity in *in vitro* mutagenicity assay.
9602869	ISO 10993-4	*In Vitro* Mammalian Cell Micronucleus Test in Human Peripheral Blood Lymphocytes for OcuPair adhesive hydrogel	OcuPair adhesive pre-hydrogel solution and hydrogel extracts did not demonstrate hematotoxicity or genotoxicity.
3101-002	ISO 10993-11	Pyrogenicity test of OcuPair by intravenous administration in Dutch belted rabbits	OcuPair adhesive pre-hydrogel solution or hydrogel extracts did not cause total increase in body temperatures in 3-hour observation period and were acceptable within USP limits concluding that it is not pyrogenic.

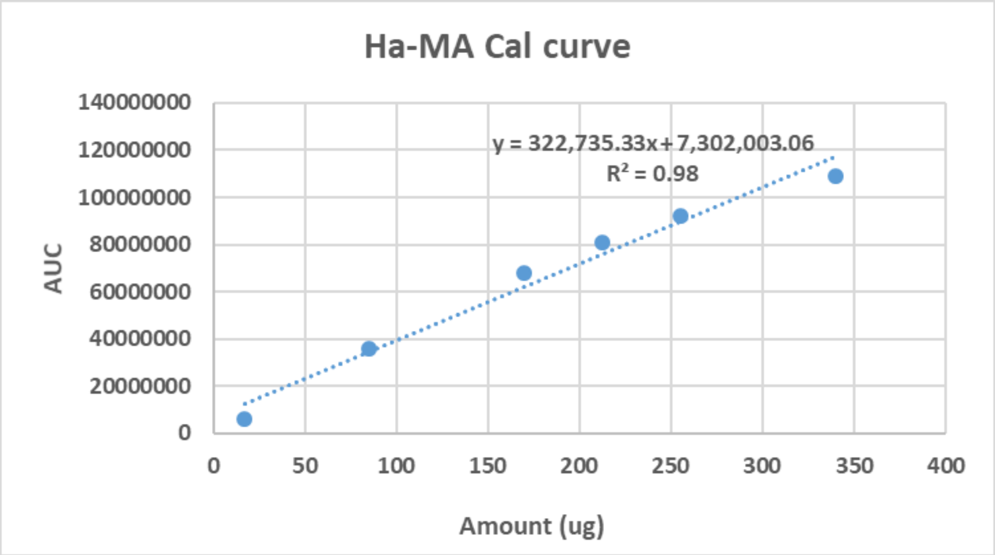

Figure S13: Calibration graph for HA-MA using HPLC analysis.

Clinical Parameters	Scores
**Anterior chamber formation**	0 = no visible chamber, 1 = shallow chamber (30-50% depth), 2 = mild shallow chamber (50-80% depth), 3 = full chamber (>80% depth)
**Corneal opacity**	0 = no opacity and completely clear, 1 = slight haze with iris and lens visible, 2 = moderately opaque, iris and lens detectable, 3 = severely opaque with iris and lens hardly visible.
**Conjunctival chemosis**	0 = no chemosis, 1+ = mild chemosis, 2+ = moderate chemosis, 3+ = severe chemosis
**Corneal epithelial edema**	0 = no edema, 1+ = mild edema, 2+ = moderate edema, 3+ = severe edema
**FLARE (corneal and anterior chamber inflammation)**	0 = no flare, 1+ = faint flare, 2+ = moderate flare (iris and lens details are clear), 3+ = marked flare (iris and lens details are hazy), 4+ = intense flare (with fibrin exudate)

Table S5: Clinical parameters and their grading score system

Legends for videos

Video 1: Rabbit eyeball with full-thickness linear (6-8mm) wound sealed using OcuPair adhesive hydrogel.

Video 2: Rabbit eyeball with full-thickness stellate wound sealed using OcuPair adhesive hydrogel.

Video 3: Peeling of adhesive hydrogel bandage from the corneal surface using surgical forceps.
